# Association Among Sperm Adiponectin, DNA Fragmentation, Oxidative Stress and Metabolites in Male Infertility

**DOI:** 10.3390/antiox14121427

**Published:** 2025-11-27

**Authors:** Rosamaria Militello, Giulia Traini, Gabriella Pinto, Tania Gamberi, Simone Luti, Sara Marchiani, Anna Illiano, Linda Vignozzi, Angela Amoresano, Alessandra Modesti

**Affiliations:** 1 Department of Biomedical, Experimental and Clinical Sciences “Mario Serio”, University of Florence, 50134 Florence, Italy; rosamaria.militello@unifi.it (R.M.); giulia.traini@unifi.it (G.T.); tania.gamberi@unifi.it (T.G.); simone.luti@unifi.it (S.L.); sara.marchiani@unifi.it (S.M.); linda.vignozzi@unifi.it (L.V.); 2 Department of Chemical Sciences, University of Naples Federico II, 80126 Naples, Italy; gabriella.pinto@unina.it (G.P.); anna.illiano@unina.it (A.I.); angela.amoresano@unina.it (A.A.)

**Keywords:** oxidative stress, male infertility, adiponectin

## Abstract

Infertility is a widespread global problem, with a male factor contributing to approximately 40–50% of cases. Several studies have investigated the involvement of adipokines in reproductive functions, but only a few have investigated their role in the male reproductive component. Collectively, adipokines are present in human sperm and most of them are expressed in the male genital tract. Some authors report that adiponectin, in contrast with other adipokines such as resistin or chemerin, has a positive effect on spermatogenesis. Although the pathophysiological role of adipokines in sperm is not yet fully understood, they could influence sperm functionality and could be potential biomarkers of male fertility. High levels of sperm DNA fragmentation have been associated with several adverse reproductive outcomes, although studies have shown conflicting results. Another critical factor in male infertility is oxidative stress, which negatively affects sperm function and viability, also because it triggers DNA alterations, lipid peroxidation and alterations in protein expression, compromising fertilization potential. To better understand the correlation between sperm DNA fragmentation, adiponectin and oxidative stress and their role in clinical practice, we evaluated these parameters in the seminal plasma of males who presented to the infertility study center of Careggi University Hospital of Florence. By accurately evaluating these parameters and their possible correlation, it will be possible to personalize treatment for individual patients.

## 1. Introduction

Approximately 50% of cases of infertility can be attributed to a male factor, alone or in combination with a female factor. In 30% of men suffering from infertility, an imbalance between reactive oxygen species (ROS) and antioxidant defenses is considered one of the causes of idiopathic male infertility [1,2]. Among other causes of infertility, many studies report the involvement of pro-inflammatory and inflammatory cytokines in male infertility. Some authors have evaluated the role of cytokines in regulating spermatogenesis [3,4]. Increased oxidative stress is related to inflammation, and much research has been conducted to understand the interactions among oxidative stress, inflammation and adiponectin [4]. In human folliculogenesis, there is a direct correlation between circulating adiponectin and the number of oocytes during in vitro fertilization. In a previous paper we investigated the correlation between total adiponectin levels and oxidative stress in the serum and follicular fluid (FF) of women who have undergone in vitro fertilization. We suggested that an increase in FF adiponectin concentration and a coincident decrease in antioxidant defenses might be involved in infertility [5]. The association between leptin and adiponectin and obesity and insulin resistance is well documented, and this association with male fertility has become evident in the last decade. The impact of these adipokines on male gonads appears to be indirect, given the conflicting results of animal and human studies [6].

Moreover, another cause that can compromise male fertility is sperm DNA fragmentation, which refers to breaks or damage in the genetic material of sperm [7]. A sperm DNA fragmentation test, which examines the genetic integrity of sperm, is an advanced test useful for supplementing a spermiogram [8]. However, it is important to distinguish among the percentage of sperm DNA fragmentation: values below 20% are considered normal, values between 20 and 30% indicate heterogeneous sperm and values above 30% indicate a significant alteration, potentially linked to infertility or recurrent miscarriages [9].

In previous study, we identified two distinct sperm populations based on their staining intensity with the dye propidium iodide (PI): PI Brighter and PI Dimmer [10]. Furthermore, only the PI Brighter population was able to distinguish between fertile and subfertile men, indicating that DNA damage in this population is better associated with reproductive outcomes [11].

To understand the correlation between sperm DNA fragmentation, adiponectin, several metabolites and their role in clinical practice, we evaluated these parameters in the seminal plasma of male partners of infertile couples attending the infertility study center of Careggi University Hospital of Florence. By accurately evaluating these parameters and their possible correlations, it will be possible to diagnose male infertility more effectively and therefore personalize treatment for individual patients.

## 2. Materials and Methods

Unless specified, all reagents were obtained from Merck (Rahway, NJ, USA), the LP Sperm Test and Anti-Ox Sperm Test were obtained from Diacron International srl (Grosseto, Italy), the Adiponectin ELISA Assay from Mediagnost (Reutlingen, Germany) and the Protein carbonyl content assay kit from Abcam (Cambridge, UK).

### 2.1. Sample Collection and Semen Analysis

Semen samples were collected from male subjects undergoing routine semen analysis for couple infertility at the Andrology Laboratory of Careggi University Hospital of Florence. The local Ethical Committee Comitato Etico Area Vasta Centro (CEAVC, protocol n. 16764_bio) approved the study. The only inclusion criterion was the obtainment of signed informed consent to use the remaining semen after completion of the analysis. Semen analysis was performed according to the World Health Organization [12] criteria using a phase-contrast microscope equipped with a heated plate maintained at 37 °C (Nikon Eclipse Ci, Nikon Europe B.V., Amstelveen, The Netherlands). After complete liquefaction of the samples, semen volume was measured. Sperm concentration was assessed using a Neubauer counting chamber. Motility was evaluated by analyzing at least 200 spermatozoa per sample, which were classified into four categories: rapid progressive, slow progressive, non-progressive and immotile. Viability was determined by eosin staining, counting at least 200 cells and distinguishing between viable and non-viable sperm. Morphology was assessed using Diff-Quik staining, with at least 200 sperm categorized as having either normal or abnormal morphology.

### 2.2. Sperm DNA Fragmentation Assessment

Sperm DNA fragmentation was evaluated using the TUNEL (Terminal deoxynucleotidyl transferase dUTP nick end labeling) assay in combination with propidium iodide (PI) staining, as previously described by Muratori et al. [10]. Samples were analyzed using flow cytometry (FACScan flow cytometer, BD Biosciences, San Jose, CA, USA), and the percentage of TUNEL-positive sperm was quantified in PI Brighter, PI Dimmer and the total sperm populations.

Samples were divided into two groups based on interquartile values of total DNA fragmentation established in fertile men. High sperm DNA fragmentation (HsDF) group included samples with a DNA fragmentation greater than 42%, whereas Low sperm DNA fragmentation (LsDF) group included those with a DNA fragmentation lower than 23%.

### 2.3. Oxidative Stress Measurement

Levels of oxidative stress on seminal plasma were measured using two colorimetric tests: the LP Sperm Test and the Anti-Ox Sperm Test, obtained from Diacron International s.r.l (Grosseto, Italy). The LP Sperm Test is a colorimetric test useful for studying male infertility and inflammatory prostate conditions. It evaluates the lipid peroxidation state, which is an important indicator of oxidative stress. The test is based on the ability of peroxides to oxidize Fe^2+^, to Fe^3+^, which then binds to a chromogenic mix, resulting in a color that is directly proportional to the lipoperoxides in the sample. The test was performed by adding 10 µL of chromogen to the chromogenic mix and incubating for 5 min at 37 °C. The sample was then centrifuged for 2 min and measured at a wavelength of 505 nm.

The Anti-Ox test evaluates the state of antioxidant defenses and/or the effectiveness of specific antioxidant treatments in the study of male infertility. Exogenous antioxidants include Vitamin C, Vitamin E, polyphenols, and endogenous antioxidants include thiol groups -SH, Uric Acid and albumin. The test is based on the ability of antioxidants to reduce ferric ion to ferrous ion, and the intensity of color is proportional to the quantity of antioxidants in the sample. In brief, 10 µL of samples were added to the reagent and incubated for 5 min at 37 °C and read at 505 nm. All measurements were performed using a free radical analyzer system (FREE Carpe Diem, Diacron International s.r.l) that included a spectrophotometric device reader and a thermostatically regulated mini-centrifuge. And the measurement kits were optimized to the FREE Carpe Diem System, according to the manufacturer’s instructions.

### 2.4. Total Protein Oxidation Measurement

Levels of total protein oxidation were measured using the Protein carbonyl content assay kit (ab126287, Abcam). The assay provides a simple method of quantifying carbonyls groups in protein samples, as protein carbonyl groups are biomarkers of oxidative stress. The assay protocol is based on the reaction of DNPH with protein carbonyls. DNP hydrazones formed in this reaction are easily quantifiable at 375 nm absorbance.

### 2.5. Adiponectin Measurement

Total adiponectin levels were detected using a specific commercial enzyme-linked immunosorbent assay kit (E09, Mediagnost, Reutlingen, Germany). Seminal plasma samples (10 µL) were diluted in 300 µL of dilution buffer and the assay was performed according to manufacturer-recommended procedures. The sensitivity provided by the manufacturer is approximately less than 0.27 ng/mL, with a detection range from 0.27 to 31000 µg/L.

### 2.6. Liquid Chromatography-Multiple Reaction Monitoring/Mass Spectrometry Analysis (LC-MRM/MS)

#### 2.6.1. Sample Preparation

One hundred µL of pooled seminal plasma samples (four pools for each group, HsDF and LsDF) were subjected to a protocol of metabolite extraction by adding four volumes of cold methanol and by centrifuging at 12,000 rpm for 20 min. An aliquot of supernatant was analysed by using liquid chromatography coupled with tandem mass spectrometry (LC-MS/MS) in Multiple Reaction Monitoring (MRM) ion mode for the targeted analyses.

#### 2.6.2. LC-MRM/MS Analysis

The analyses were performed using a Sciex 5500 QTrap mass spectrometry system equipped with exionLCTM. Chromatographic separation was performed using a Kinetex C18 column (5 μm particle size, length 100 × internal diameter 2.1 mm; Phenomenex, Torrance, CA, USA) with a flow rate of 0.2 mL/min, maintained at 40 °C. The separation was obtained using a linear gradient from 2% to 98% of mobile phase B on a total run time of 8 min with a flow rate of 0.2 mL/min. Mobile phase A was composed of water with 0.1% formic acid and 5 mM ammonium formate, while mobile phase B consisted of acetonitrile containing 0.1% formic acid. A linear gradient was applied, ranging from 2% to 98% of mobile phase B.

Electrospray ionization (ESI) was operated in positive ion mode for the detection of amino acids and steroid hormones, and in negative ion mode for organic acids. The ESI source was configured with the following parameters: a curtain gas pressure of 20 psi, a medium collision gas pressure, an ion spray voltage of 5.5 kV and a source temperature of 500 °C. Instrumental parameters of the MRM method, including precursor and product ion mass-to-charge ratios (*m*/*z*), collision energies and dwell times, were optimized based on previously published data from the literature [5,13]. The raw data were imported into Skyline software 25.1 [14] to facilitate the visualization of chromatographic peaks corresponding to all monitored transitions and to extract peak area values from each biological sample for subsequent quantitative analysis.

### 2.7. Statistical Analysis

Data are presented as means +/− standard deviation (SD). Statistical analysis was performed by unpaired test using GraphPad Prism 8. The test compared every mean with every other mean. Pearson correlation analysis was performed among data of levels of oxidative stress in seminal plasma and semen characteristics’ value. Significance was defined as *p*-value (*p*) < 0.05. Principal component analysis (PCA) was performed on metabolomics data by MetaboAnalyst 6.0 stat module (https://www.metaboanalyst.ca/) using the default parameters. The differentially expressed metabolites were identified by performing the one-way ANOVA test, followed by Tukey’s multiple comparisons test, using GraphPad Prism 8. Data are shown as means +/− standard deviation (SD) from at least three experiments. *p* < 0.05 were considered statistically significant (* *p* < 0.05; ** *p* < 0.01; *** *p* < 0.001; **** *p* < 0.0001).

## 3. Results

### 3.1. Human Seminal Plasma Characteristics

In Table 1 we report the semen characteristics of the 20 subjects included in the study. For our analysis we divided the samples according to the percentage of DNA fragmentation. According to several authors, an index lower than 15% is generally considered optimal [9,15].

**Figure 1:**
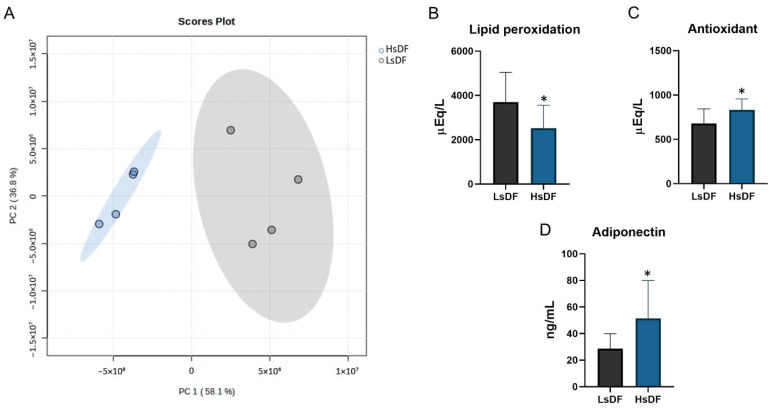
(**A**) Principal component analysis performed by Metaboanalist 6.0 stat module using the default parameters. The statistical significances of the group patterns were evaluated using the Permutational Multivariate Analysis of Variance (PERMANOVA). The distributions were computed using the Euclidean distance based on the Principal Components (PCs). Seminal plasma oxidative stress measurements in samples with low sperm DNA fragmentation (LsDF) and high sperm DNA fragmentation (HsDF). (**B**) Lipid peroxidation state, (**C**) antioxidant capacity and (**D**) levels of adiponectin. Measurements were carried out on each sample separately. Statistical analysis was performed by unpaired test using GraphPad Prism 8. Results were reported as means +/− standard deviation (SD). Significance was defined as *p*  < 0.05 and indicated with an asterisk (*).

For this reason, we decided to divide the semen samples into two groups: HsDF (*n* = 10; from 42.2 to 92.7 DNA fragmentation index) and LsDF (*n* = 10; from: 8.8 to 22.7 DNA fragmentation index). The HsDF group exhibited markedly higher values for both total fragmentation (50.7 ± 14.9 vs. 16.7 ± 4.3; **** *p* < 0.0001) and brighter fragmentation (24.9 ± 6 vs. 9.9 ± 2.3; **** *p* < 0.0001). Principal component analysis (PCA), performed with MetaboAnalyst and shown in Figure 1A, clearly separates the two groups.

The two groups, LsDF and HsDF, showed no significant differences in mean age of the participants (39.1 ± 5.1 years vs. 39.2 ± 6.9 years), body mass index (25.5 ± 2.8 vs. 23.8 ± 3.1) and in other standard semen parameters reported in Table 1. However, as expected, significant differences emerged in sperm motility. Sperm motility is the ability of sperm to move efficiently: according to movement and velocity, spermatozoa can be graded as progressively motile, non-progressively motile and immotile sperm [16]. Progressive motile sperm refers to the percentage of sperm that move actively in a straight line or large circles, determining a successful navigation through the female reproductive tract with better chances of natural conception, as these sperm are more likely to reach and fertilize the oocyte. Non-progressive motile sperm includes spermatozoa that move irregularly or only laterally with their heads, without forward progression [16]. Total mobility is the sum of progressive and non-progressive motile spermatozoa. Therefore, assessing both total motility and progressive motility provides valuable insights into sperm quality and fertilizing potential. The HsDF group showed a significantly reduced progressive motility (43 ± 16.5% vs. 60.6 ± 8.2%; ** *p* < 0.01). Total motility was also significantly lower in the HsDF group (49.6 ± 16.1% vs. 67.5 ± 7.1%; ** *p* < 0.01) compared to LsDF.

### 3.2. Relationship Between Sperm DNA Fragmentation and Seminal Oxidative Level in LsDF and HsDF Groups

DNA fragmentation is one of the main elements influencing the quality of semen and consequently the fertilizing ability. In this scenario, oxidative stress plays a pivotal role in the molecular mechanisms causing DNA damage [17]. Therefore, we investigated the relationship between sperm DNA fragmentation, seminal lipid peroxidation and antioxidant levels. Surprisingly, we found, as reported in Figure 1B, a low level of lipoperoxidation in HsDF samples in comparison to LsDF samples. As evident in the figure, this value is −47.22% compared to LsDF (*p* = 0.0398); however, in contrast to the data reported above, we found a high capacity for protection from oxidation, because in HsDF samples, the antioxidant levels show a significant increase of 22.52% (*p* = 0.0292), as shown in Figure 1C. We evaluated the total protein oxidation in HsDF and LsDF samples, since proteins are molecules sensitive to irreversible modification that could be caused by oxidation. The statistical analysis performed demonstrated no significant difference between the two groups.

### 3.3. Seminal Adiponectin Levels in LsDF and HsDF Groups

Adiponectin has been claimed to have an important role in male reproduction and infertility. Since the role of adiponectin and its correlation with sperm DNA fragmentation is still unclear and controversial, we evaluated whether adiponectin level in seminal plasma may be associated with sperm DNA fragmentation, and the results reported in Figure 1D show that HsDF samples have a significant higher level of this adipokine, with an increase of 79.36% compared to LsDF samples (*p* = 0.0425).

### 3.4. Correlation Among Sperm DNA Fragmentation and Semen Parameters in LsDF and HsDF Groups

In the LsDF group we observed no correlation between total sperm DNA fragmentation and other parameters.

**Figure 2:**
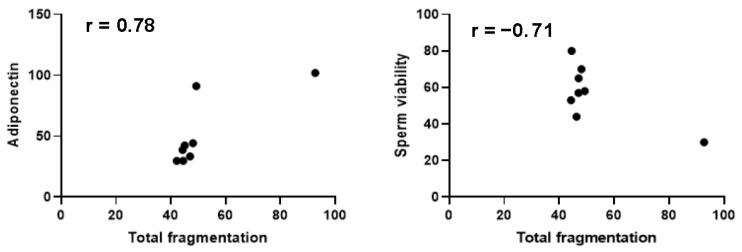
Pearson correlation analysis in the high sperm DNA fragmentation group. A confidence interval of 95% was selected. Significance was defined as *p* < 0.05.

In the HsDF group, as shown in Figure 2, a positive correlation was found between sperm DNA fragmentation and seminal adiponectin level (r = 0.78; *p* = 0.023), and in addition, this group shows a negative correlation with sperm viability (r = −0.71; *p* = 0.048).

### 3.5. Metabolic Profile in the LsDF and HsDF Groups

**Figure 3:**
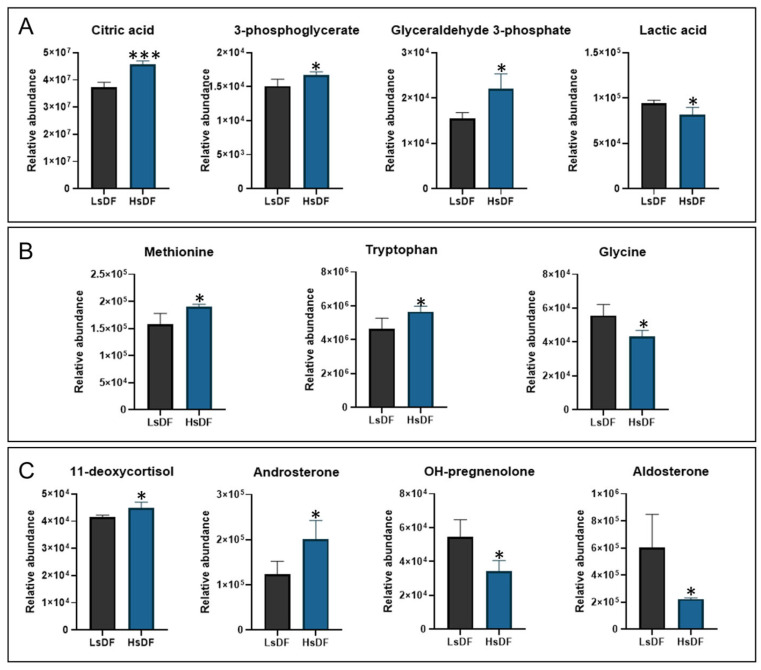
Metabolomic profile of seminal plasma (**A**) organic acid, (**B**) amino acids and (**C**) steroid hormones. Histograms represent metabolites whose relative abundance was statistically different (* *p* < 0.05; *** *p* < 0.001) between samples with low sperm DNA fragmentation (LsDF) and high sperm DNA fragmentation (HsDF). Statistical analysis was performed by unpaired test using GraphPad Prism 8. Results were reported as means +/− standard deviation (SD).

To identify metabolites as possible biomarkers associated with the degree of sperm DNA fragmentation, we investigated the differences in sperm metabolic patterns in the samples. HsDF and LsDF samples were subjected to metabolomics analysis using GC-MS and LC-MRM/MS. From the analysis we identified 50 metabolites (see Supplementary Table S1). Twelve of these were differentially abundant between HsDF and LsDF groups. These metabolites, reported in Figure 3, were divided into three groups: organic acids, amino acids and steroid hormones. Organic acids shown in Figure 3A: Citric acid (+22.56%, *p* = 0.0003), 3-phosphoglycerate (+10.94%, *p* = 0.0232) and glyceraldehyde 3-phosphate (+42.59%, *p* = 0.0106) were significantly increased in the HsDF group compared to the LsDF group. The only organic acid found to be decreased in the HsDF group was lactic acid (–15.84%, *p* = 0.0256). Amino acids shown in Figure 3B: Methionine (+20.75%, *p* = 0.02) and tryptophan (+22.22%, *p* = 0.0274) were elevated in the HsDF group, while glycine was reduced (–28.6%, *p* = 0.0165), compared to LsDF. Steroid hormones shown in Figure 3C: 11-deoxycortisol (+8%, *p* = 0.0295) and androsterone (+62.88%, *p* = 0.0218) were increased in the HsDF group relative to the LsDF group. In contrast, OH-pregnenolone (–58.65%, *p*= 0.0145) and aldosterone (–170.57%, *p*= 0.0211) were significantly decreased.

### 3.6. Correlation Among Hormones and Metabolites in LsDF and HsDF Groups

**Figure 4:**
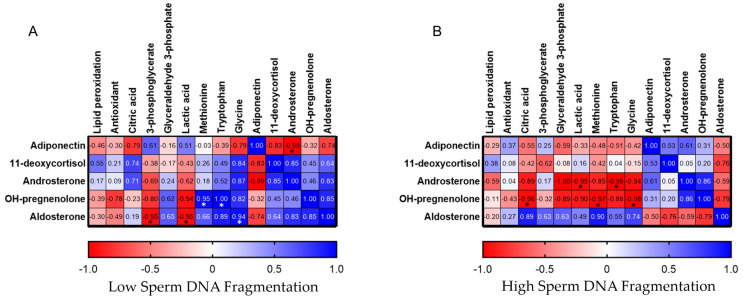
Heatmap of Pearson correlation coefficients among adiponectin and steroid hormones with oxidative stress measurements and other metabolites. (**A**): LsDF; Panel **(B**): HsDF. Colors intensity indicates the strength of the correlation, with red representing positive correlations and blue representing negative correlations.* Significance was defined as *p* < 0.05.

A heatmap of Pearson correlation coefficients reported in Figure 4 visually displays the strength and direction of linear relationships between hormones and other metabolites identified for LsDF and HsDF seminal plasma. In the heatmap, the red colors indicate a positive linear relationship and the blue a negative. As reported in Figure 4A, adiponectin is negatively correlated with androsterone (r = −0.99; *p* = 0.011), a steroid hormone with weak androgenic activity in the LsDF group but showing the reverse behavior in the HsDF group (r = 0.61; *p* = 0.39). As shown in figure, adiponectin in the LsDF group is also negatively correlated with 11-deoxycortisol (r = −0.99; *p* = 0.172), a cortisol precursor, and in this case, it shows an inverse behavior in the HsDF group (r = 0.53; *p* = 0.468). Pregnenolone, in the HsDF group, shows negative correlations with methionine (r = −0.97; *p* = 0.03), glycine (r = −0.98; *p* = 0.015) and tryptophan (r = −0.88, *p* = 0.12), and again the trend is opposite for the LsDF group (methionine r = 0.95, *p* = 0.965; glycine r = 0.82, *p* = 0.184; tryptophan r = 1, *p* = 0.003), as shown in Figure 4B.

### 3.7. Metabolomic Pathways Analysis for LsDF and HsDF Groups

**Figure 5:**
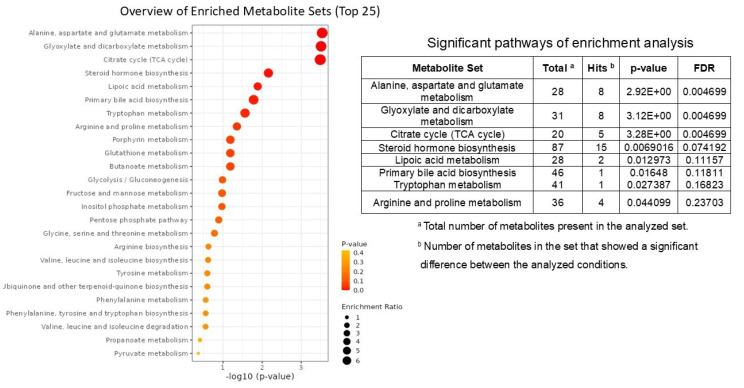
Representation of enrichment metabolic pathways in high sperm DNA fragmentation (HsDF) and low sperm DNA fragmentation (LsDF) samples obtained by MetaboAnalist 6.0 with no modifications. Circle size represents the number of metabolites in each set, and color indicates statistical significance (adjusted *p*-value). The analysis was performed used MetaboAnalyst 6.0 and KEGG was selected as the metabolite set library. Pathways reported in the table were selected based on *p* < 0.05.

To better understand these metabolomic results and highlight relevant metabolic pathways, an enrichment analysis was performed using MetaboAnalyst 6.0. A list of compound names with concentrations was uploaded and KEGG was selected as the metabolite set library. The results of the enrichment analysis are reported in the dot plot in Figure 5, in which the size of the circles per metabolite set represents the Enrichment Ratio and the color represents the *p*-value. Pathway selection was based on *p*-value < 0.05 and results are shown in the table in Figure 5. The data pointed out an enrichment of pathways involved in amino acid metabolism (alanine, aspartate and glutamate), the citrate cycle and steroid hormone biosynthesis.

## 4. Discussion

Adiponectin is an adipokine predominantly secreted by adipose tissue, recognized for its anti-inflammatory and antioxidant properties. Its circulating levels are inversely correlated with adiposity and are typically reduced in individuals affected by obesity and metabolic syndrome [18]. This reduction contributes to a pro-oxidative systemic environment, which may negatively affect various physiological processes, including reproductive function. In a previous paper, we investigated the correlation between total adiponectin levels and oxidative stress in the serum and follicular fluid (FF) of women who had undergone in vitro fertilization. We suggested that an increase in FF adiponectin concentration and a coincident decrease in antioxidant defenses might be involved in infertility [5]. Although the direct role of adiponectin within seminal plasma remains to be fully elucidated, its systemic antioxidant activity suggests a potential protective effect against oxidative damage also on spermatozoa. Oxidative stress is a well-established contributor to male infertility, impairing sperm motility, morphology and DNA integrity [19].

The hypothesis of a direct role of adiponectin in testicular function through local signaling pathways is supported by the presence of adiponectin receptors (AdipoR1 and AdipoR2) in testicular tissue, including Leydig and Sertoli cells [20].

The aim of this study was to clarify the correlation of adiponectin with sperm oxidative damage to determine its potential utility as a biomarker or adjunctive treatment in clinical andrological practice. To this end, adiponectin level was measured alongside antioxidant capacity and lipid peroxidation levels in the seminal plasma of male partners from infertile couples. The patients were divided in two groups according to the different level of sperm DNA fragmentation: low DNA fragmentation (HsDF) and high DNA fragmentation (HsDF). The results revealed that seminal fluid from individuals with HsDF exhibited significantly higher adiponectin concentrations, increased antioxidant capacity and reduced lipid peroxidation. These findings suggest the activation of a compensatory mechanism aimed at mitigating oxidative damage. The high adiponectin level could create a more favorable environment for sperm survival by limiting oxidative and inflammatory damage. In practice, the body attempts to protect spermatozoa that are still viable (or less severely damaged) and, where possible, repair existing damage. The observed reduction in lipid peroxidation may therefore be a direct consequence of this enhanced antioxidant defense. Despite this antioxidant activity and adiponectin levels, high sperm DNA fragmentation persists. This suggests that the underlying causes of DNA damage could not be primarily attributable to local oxidative stress. The scenario appears to be more complex and is likely linked to multiple factors. In other words, DNA fragmentation might also occur during spermatogenesis, sperm transport through the seminiferous tubules and the epididymis, or might be affected by the unbalance of circulating molecules like hormones, cytokines and nutrients as well as the exposure to environmental toxicants that could initiate or influence the antioxidant pathway within the testes [21].

Our hypothesis, of a compensatory response associated with a higher adiponectin level, is further supported by metabolomic analysis. The obtained results revealed alterations in key energy-related metabolites, consistent with an adaptive antioxidant upregulation in the presence of severe sperm DNA damage. The observed accumulation of glyceraldehyde-3-phosphate (G3P), a key glycolytic intermediate, alongside reduced lactate levels, leads to an impairment of glycolytic flux. Concurrently, elevated citric acid levels may reflect a compensatory shift toward mitochondrial oxidative metabolism. This imbalance indicates an inefficient energy production pathway, which can lead to reduced ATP availability, compromising sperm function and contributing to DNA fragmentation due to insufficient energy for chromatin maintenance and repair.

Metabolomic profiling also revealed elevated methionine and tryptophan levels, alongside reduced glycine, suggesting disrupted methylation and redox balance. Methionine, a precursor of S-adenosylmethionine (SAM), may reflect compensatory methylation activity aimed at genomic stabilization [22], while decreased glycine points to impaired antioxidant defense via reduced glutathione synthesis [23]. These amino acid shifts, combined with impaired glycolysis and mitochondrial stress, support a multifactorial metabolic dysfunction underlying sperm DNA fragmentation beyond localized oxidative stress [24]. Systemic inflammation or stress adaptation should provide for a decrease in the level of tryptophan, as it will be metabolized to kynurenine metabolites, which will help cope with inflammation and associated oxidative stress. On the contrary, we find an increase in the tryptophan level and a concomitant reduction in pregnenolone, a precursor of testosterone; several research indicates that high levels of tryptophan can lead to a reduction in testosterone, negatively affecting testicular steroidogenesis. The interaction between high tryptophan levels and low testosterone could negatively impact sperm quality [25]. In fact, the metabolomics analysis revealed a different steroid hormones profile in the seminal plasma of HsDF spermatozoa. In this seminal plasma, we found higher levels of deoxicortisol and androsterone, associated with a decrease in pregnenolone and aldosterone. Deoxycortisol is a precursor of cortisol whose increase is generally associated with chronic stress such as oxidative stress in the testes, which is a major known cause of sperm DNA damage/fragmentation [26]. Direct correlation studies for androsterone in seminal plasma with sperm count, motility or morphology are limited or inconclusive in the broader literature. Aldosterone is an inflammatory and fibrotic agent. Elevated levels in the reproductive tract environment could potentially increase oxidative stress by promoting local inflammation. Contrary to this observation, in the seminal plasma of HsDF spermatozoa we found low levels of pregnenolone and aldosterone. Pregnenolone is the precursor of almost all other steroid hormones (progesterone and androgens like testosterone, estrogens, cortisol and aldosterone). It is produced in the adrenal glands and, crucially for male fertility, in the testicles. This result suggests a link with the observed tryptophan trend. In summary, the correlation suggests that high tryptophan levels can contribute to a reduction in testosterone levels by disrupting the normal hormonal signaling pathway between the brain, pituitary gland and testes [25]. The interaction between high tryptophan levels and low testosterone could negatively impact sperm quality.

Aldosterone is produced by the adrenal glands and it is involved in regulating salt, water and blood pressure. Its receptors are also present in the male reproductive tract, suggesting a local role in regulating the electrolytic environment of the seminal plasma. The reduced presence of this hormone could be an indicator of impaired testicular function or an unfavorable microenvironment that is prone to the defects (like increased oxidative stress) leading to DNA fragmentation.

## 5. Conclusions

This study investigated the role of adiponectin in seminal plasma and its correlation with sperm DNA fragmentation, finding that its action is part of a complex, multifactorial response to severe sperm damage. Our results suggest that high sperm DNA fragmentation (HsDF) is primarily linked, not only to acute local oxidative stress, but probably also to intrinsic defects in spermatogenesis and metabolic dysfunctions that overwhelm local antioxidant defenses. In detail, we found significantly higher levels of Adiponectin and increased antioxidant capacity, coupled with reduced lipid peroxidation. This could be interpreted as an activated compensatory mechanism that, despite its strength, is insufficient to prevent DNA damage, suggesting that the primary cause of HsDF is non-local.

In conclusion, this work represents a preliminary study designed to establish the initial correlations between adiponectin levels, oxidative stress, DNA fragmentation and several amino acids in seminal fluid. Further studies will be conducted to confirm the proposed hypotheses and to elucidate the underlying molecular mechanisms. Indeed, it will be required to investigate adiponectin receptor signaling activity in testicular cells, validate the metabolite and hormone findings in a larger, independent cohort and perform mechanistic studies isolating the link between specific metabolic faults and DNA repair failure.

## Figures and Tables

**Table 1:** Mean ± SD of age, BMI and basal semen characteristics of subjects included in the two groups of the study: low sperm DNA fragmentation (LsDF) and high sperm DNA fragmentation (HsDF).

	LsDF	HsDF
**Age (years)**	39.1 ± 5.1	39.2 ± 6.9
**BMI ^a^ (kg/m^2^)**	23.8 ± 3.1	25.5 ± 2.8
**Total fragmentation**	16.7 ± 4.3	50.7 ± 14.9 ****
**Brighter fragmentation**	9.9 ± 2.3	24.9 ± 6 ****
**Semen volume (mL)**	4.4 ± 1.8	5.6 ± 2.5
**pH**	7.3 ± 1.5	7.6 ± 0.2
**Sperm concentration (×10^6^/mL)**	66.4 ± 50.8	74.2 ± 67.5
**Sperm total count (×10^6^/ejaculate)**	277.9 ± 209.9	361.9 ± 379
**Sperm normal morphology (%)**	4.5 ± 2.8	2.9 ± 2
**Sperm progressive motility (%)**	60.6 ± 8.2	43 ± 16.5 **
**Sperm total motility (%)**	67.5 ± 7.1	49.6 ± 16.1 **
**Sperm viability (%)**	74 ± 8.7	57.1 ± 15.5

^a^ Body mass index. Data are reported as mean ± Standard deviation (SD). Unpaired *t*-test was performed by GraphPad Prism 8.0 software between LsDF and HsDF (** *p* < 0.01; and **** *p* < 0.0001).

## Data Availability

The authors confirm that the data supporting the findings of this study are available within the article and its Supplementary Materials.

## Supplementary Materials

The following are available online at https://www.mdpi.com/article/10.3390/antiox14121427/s1, Table S1: List of identified metabolites through GC–MS.
