# Supplementary material for: Association Among Sperm Adiponectin, DNA Fragmentation, Oxidative Stress and Metabolites in Male Infertility
Source: Antioxidants (Basel). 2025 Nov 27;14(12):1427. doi: 10.3390/antiox14121427 (PMC12729378; doi:10.3390/antiox14121427)
Supplement: Supplementary file 1 [file antioxidants-14-01427-s001.zip › antioxidants-3976153-supplementary.pdf]

Supplementary Table S1. List of identified metabolites through GC–MS.

Name	HMDB [a]	PubChem	KEGG ID [b]
Citric acid	HMDB0000094	311	C00158
Isocitric acid	HMDB0000193	1198	C00311
3-Phosphoglyceric acid	HMDB0000807	724	C00597
Glucose 6-phosphate	HMDB0001401	5958	C00092
Glyceraldehyde 3-phosphate	HMDB0001112	439168	C00118
Succinic acid	HMDB0000254	1110	C00042
Malic acid	HMDB0000156	222656	C00149
Aconitic acid	HMDB0247961	309	-
D-Glucose	HMDB0000122	5793	C00031
Pyruvic acid	HMDB0000243	1060	C00022
Fructose 1,6-bisphosphate	HMDB0001058	10267	C00354
Fructose 1-phosphate	HMDB0001076	10400369	C01094
D-Ribose 5-phosphate	HMDB0001548	440101	C00117
Lactic acid	HMDB0000190	107689	C00186
Glycine	HMDB0000123	750	C00037
L-Alanine	HMDB0000161	5950	C00041
Serine	HMDB0000187	5951	C00065
Proline	HMDB0000162	145742	C00148
L-Valine	HMDB0000883	6287	C00183
L-Threonine	HMDB0000167	6288	C00188
Isoleucine	HMDB0000172	6306	C00407
L-Aspartic acid	HMDB0000191	5960	C00049
Lysine	HMDB0000182	5962	C00047
Glutamic acid	HMDB0000148	33032	C00025
Methionine	HMDB0000696	6137	C00073
Histidine	HMDB0000177	6274	C00135
Phenylalanine	HMDB0000159	6140	C00079
L-Arginine	HMDB0000517	6322	C00062
L-Tyrosine	HMDB0000158	6057	C00082
L-Cysteine	HMDB0000574	5862	C00097
L-Tryptophan	HMDB0000929	6305	C00078
L-Asparagine	HMDB0000168	6267	C00152
Glutamine	HMDB0000641	5961	C00064
Cortexolone	HMDB0000015	440707	C05488
17-Hydroxyprogesterone	HMDB0000374	6238	C01176
17a-Hydroxypregnenolone	HMDB0000363	91451	C05138
21-Deoxycortisol	HMDB0004030	222803	C05497
Aldosterone	HMDB0000037	5839	C01780
Androstenedione	HMDB0000053	6128	C00280
Androsterone	HMDB0000031	5879	C00523
Corticosterone	HMDB0001547	5753	C02140
Cortisol	HMDB0000063	5754	C00735
Dihydrotestosterone	HMDB0002961	10635	C03917
Estradiol	HMDB0000151	5757	C00951

Estrone	HMDB0000145	5870	C00468
Methyltestosterone	HMDB0015655	6010	C07198
Nandrolone	HMDB0002725	9904	C07254
Pregnenolone	HMDB0000253	8955	C01953
Progesterone	HMDB0001830	5994	C00410
Testosterone	HMDB0000234	6013	C00535

[a] Human Metabolome Database

[b] Kyoto Encyclopedia of Genes and Genomes Database
